# Conserved longitudinal alterations of anti-S-protein IgG subclasses in disease progression in initial ancestral Wuhan and vaccine breakthrough Delta infections

**DOI:** 10.3389/fmicb.2022.1043049

**Published:** 2022-11-22

**Authors:** Yun Shan Goh, Siew-Wai Fong, Pei Xiang Hor, Siti Naqiah Amrun, Cheryl Yi-Pin Lee, Barnaby Edward Young, Po Ying Chia, Paul A. Tambyah, Shirin Kalimuddin, Surinder Pada, Seow-Yen Tan, Louisa Jin Sun, Mark I-Cheng Chen, Yee-Sin Leo, David C. Lye, Lisa F. P. Ng, Laurent Renia

**Affiliations:** ^1^A*STAR Infectious Diseases Labs (A*STAR ID Labs), Agency for Science, Technology and Research (A*STAR), Singapore, Singapore; ^2^National Centre for Infectious Diseases, Singapore, Singapore; ^3^Department of Infectious Diseases, Tan Tock Seng Hospital, Singapore, Singapore; ^4^Lee Kong Chian School of Medicine, Nanyang Technological University, Singapore, Singapore; ^5^Department of Medicine, Yong Loo Lin School of Medicine, National University of Singapore, Singapore, Singapore; ^6^Department of Infectious Diseases, National University Health System, Singapore, Singapore; ^7^Department of Infectious Diseases, Singapore General Hospital, Singapore, Singapore; ^8^Emerging Infectious Disease Program, Duke-NUS Medical School, Singapore, Singapore; ^9^Division of Infectious Diseases, Ng Teng Fong Hospital, Singapore, Singapore; ^10^Department of Infectious Diseases, Changi General Hospital, Singapore, Singapore; ^11^Alexandra Hospital, Singapore, Singapore; ^12^Saw Swee Hock School of Public Health, National University of Singapore, Singapore, Singapore; ^13^Yong Loo Lin School of Medicine, National University of Singapore and National University Health System, Singapore, Singapore; ^14^National Institute of Health Research, Health Protection Research Unit in Emerging and Zoonotic Infections, University of Liverpool, Liverpool, United Kingdom; ^15^Institute of Infection, Veterinary and Ecological Sciences, University of Liverpool, Liverpool, United Kingdom; ^16^School of Biological Sciences, Nanyang Technological University, Singapore, Singapore

**Keywords:** SARS-CoV-2, COVID-19, S protein, antibodies, IgG subclasses, severity, cytokines, recovery

## Abstract

**Introduction:**

COVID-19 has a wide disease spectrum ranging from asymptomatic to severe. While humoral immune responses are critical in preventing infection, the immune mechanisms leading to severe disease, and the identification of biomarkers of disease progression and/or resolution of the infection remains to be determined.

**Methods:**

Plasma samples were obtained from infections during the initial wave of ancestral wildtype SARS-CoV-2 and from vaccine breakthrough infections during the wave of Delta variant, up to six months post infection. The spike-specific antibody profiles were compared across different severity groups and timepoints.

**Results:**

We found an association between spike-specific IgM, IgA and IgG and disease severity in unvaccinated infected individuals. In addition to strong IgG1 and IgG3 response, patients with severe disease develop a robust IgG2 and IgG4 response. A comparison of the ratio of IgG1 and IgG3 to IgG2 and IgG4 showed that disease progression is associated with a smaller ratio in both the initial wave of WT and the vaccine breakthrough Delta infections. Time-course analysis revealed that smaller (IgG1 and IgG3)/(IgG2 and IgG4) ratio is associated with disease progression, while the reverse associates with clinical recovery.

**Discussion:**

While each IgG subclass is associated with disease severity, the balance within the four IgG subclasses may affect disease outcome. Acute disease progression or infection resolution is associated with a specific immunological phenotype that is conserved in both the initial wave of WT and the vaccine breakthrough Delta infections.

## Introduction

The coronavirus disease 2019 (COVID-19) is an ongoing pandemic, affecting 223 countries, with 532 million confirmed infection cases and 6.3 million fatalities to date (WHO, 2022). The disease, caused by SARS-CoV-2 (Cohen and Normile, 2020; Wu et al., 2020; Zhou P. et al., 2020), can lead to many clinical manifestations, ranging from mild to severe symptoms. Most patients have mild symptoms such as fever, cough, fatigue, anosmia, sore throat, and headache (Chen et al., 2020; Yang et al., 2020). Approximately 20% of symptomatic patients develop severe disease, with 5% progressing to critical stages, which could include respiratory failure, pneumonia, multiple organ failure and, in the most serious cases, death (Chen et al., 2020; Wu and McGoogan, 2020; Zhou F. et al., 2020). While immune responses in recovered COVID-19 patients persist for six months post-infection (Dan et al., 2021), the immune longevity can vary considerably between individuals (Chia et al., 2021). The advent of Spike (S) protein-based mRNA COVID-19 vaccines has significantly improved the COVID-19 pandemic, with both Pfizer-BioNTech (BNT162b2) and Moderna (mRNA-1,273) reporting 95% vaccine efficacy (Polack et al., 2020; Baden et al., 2021). The incidence of severe cases has greatly reduced (Selvaraj et al., 2022). However, vaccine breakthrough infections do occur, potentially due to a suboptimal anti-S-protein antibody response (Sanghavi et al., 2022). Thus, there is a critical need to better understand the humoral immunity against the S protein in both unvaccinated and vaccinated individuals to inform public health decisions on primary or booster vaccination and, possibly, treatment and prophylaxis.

We have previously found that IgG1 is the dominant IgG subclass against S protein in COVID-19 patients (Goh et al., 2021a,b). IgG1 and IgG3 induction, typically indicative of a T helper 1 (Th1) response (Kawasaki et al., 2004), are pro-inflammatory responses particularly important in protective immunity against viruses. IgG1 and IgG3 possess higher neutralization capabilities against many viruses (Hofmeister et al., 2011; Richardson et al., 2019; Walker et al., 2020) and mediate important antiviral functions, such as phagocytosis or NK cell-mediated killing of infected cells, through Fc receptors (Chung et al., 2018; Fischinger et al., 2020).

In this study, we examined the antibody profiles against S protein in unvaccinated patients with asymptomatic SARS-Cov-2 infection and with varying clinical severities of COVID-19. We also investigated whether disease severity was associated with a particular IgG subclass profile. Similarly, the antibody profiles against S protein in vaccinated individuals who have vaccine breakthrough infection, were also examined. The longitudinal antibody profiles were assessed to determine the longevity of specific immune response following infection in unvaccinated and vaccinated individuals.

## Materials and methods

### Ethics approval

The study design and protocols for symptomatic and asymptomatic COVID-19 patient were approved by National Healthcare Group Domain Specific Review Board and performed, following ethical guidelines in the approved study 2012/00917. Healthy donor samples were collected in accordance with approved studies 2017/2806 and NUS IRB 04–140. All studies were performed in accordance with the Declaration of Helsinki for Human Research. Written informed consent was obtained from all participants.

### Plasma samples

A total of 131 unvaccinated patients (symptomatic, *n* = 81 and asymptomatic, *n* = 50, Supplementary Table S1), who tested PCR-positive for SARS-CoV-2 by nasopharyngeal swab, were recruited from January to September 2020 (Amrun et al., 2020; Pung et al., 2020), and a total of 118 vaccinated individuals (Supplementary Table S1), who have vaccine breakthrough Delta infections, were recruited from April to August 2022. Samples were collected from unvaccinated patients at median 5-days (3–7 days), median 10-days (8–13 days), median 23-days (15–32 days), median 101-days (88–106 days) and median 180-days (153–199 days). For vaccine breakthrough Delta infections, samples were collected at median 1-day (1 day), median 36-days (27–53 days), median 94-days (80–113 days) and median 178-days (158–200 days). Symptomatic patients were classified into three groups based on clinical severity: mild (no pneumonia on chest radiographs (CXR) at baseline and during hospital admission), moderate (pneumonia without hypoxia), and severe (pneumonia with hypoxia (desaturation ≤94%)). For comparisons on pneumonia, patients without pneumonia include mild patients, while patients with pneumonia include moderate and severe patients. For comparisons on oxygen supply requirement, patients without oxygen supply requirement include mild and moderate patients, while patients with oxygen supply requirement include severe patients. For comparison on intensive care unit (ICU)-admission, patients without ICU admission include mild, moderate and a subset of non-ICU-admitted severe patients, and patients with ICU admission include only ICU-admitted severe patients. Asymptomatic infections were individuals with a positive SARS-CoV-2 PCR test but without any COVID-19 symptoms in the 3 months before the first test till follow-up 28 days later.

### S protein flow cytometry-based assay for antibody detection (SFB assay)

The assay was performed as previously described (Goh et al., 2021a,b). Cells expressing either ancestral Wuhan wildtype (Goh et al., 2021a,b) or Delta (Wang et al., 2021) variant S protein were seeded at 1.5 × 10^5^ cells per well in 96 well plates (ThermoFisher Scientific). Expression of the different S proteins were previously verified (Renia et al., 2022) and the amount of spike protein expressed on the cell surface was found to be similar for cell lines expressing the spike protein of both ancestral Wuhan wildtype and Delta variants (Goh et al., 2022). The cells were first incubated with plasma (1, 100) before a secondary incubation with a double stain, consisting of Alexa Fluor 647-conjugated secondary antibodies (1,500) and propidium iodide (PI (Sigma-Aldrich); 1:2500). Secondary antibodies are conjugated anti-human IgM, IgA or IgG (ThermoFisher Scientific). For assays examining IgG subclasses, the secondary incubation was with mouse anti-human IgG1 Fc (monoclonal antibody clone 2C11; ThermoFisher Scientific), IgG2 Fc (monoclonal antibody clone HP6002, BioLegend), IgG3 Fc (monoclonal antibody clone HP6047; BioLegend), or IgG4 Fc (monoclonal antibody clone HP6025; ThermoFisher Scientific). The cells were then incubated with Alexa Fluor 647-conjugated anti-mouse IgG (ThermoFisher Scientific). Cells were read on BD Biosciences LSR4 laser and analyzed using FlowJo (Tree Star), by gating on: (1) FSC-A/SSC-A to exclude cell debris, (2) FSC-A/FSC-H for single cells, (3) FSC-A/PI for live cells (PI-negative population), (4) FITC/Alexa Fluor 647 (Supplementary Figure 1). Binding is determined by the percentage of antibody-bound GFP-positive S protein-expressing cells, indicated by Alexa Fluor 647-and FITC-positive events.

### Statistical analysis

Statistical analysis was done using Prism (GraphPad). For comparing between multiple groups, Kruskal-Wallis tests and *post hoc* tests using Dunn’s multiple comparison tests were used to identify significant differences. *p* values less than 0.05 are considered significant.

## Results

### High levels of antibodies against S protein associated with disease severity

**Figure 1:**
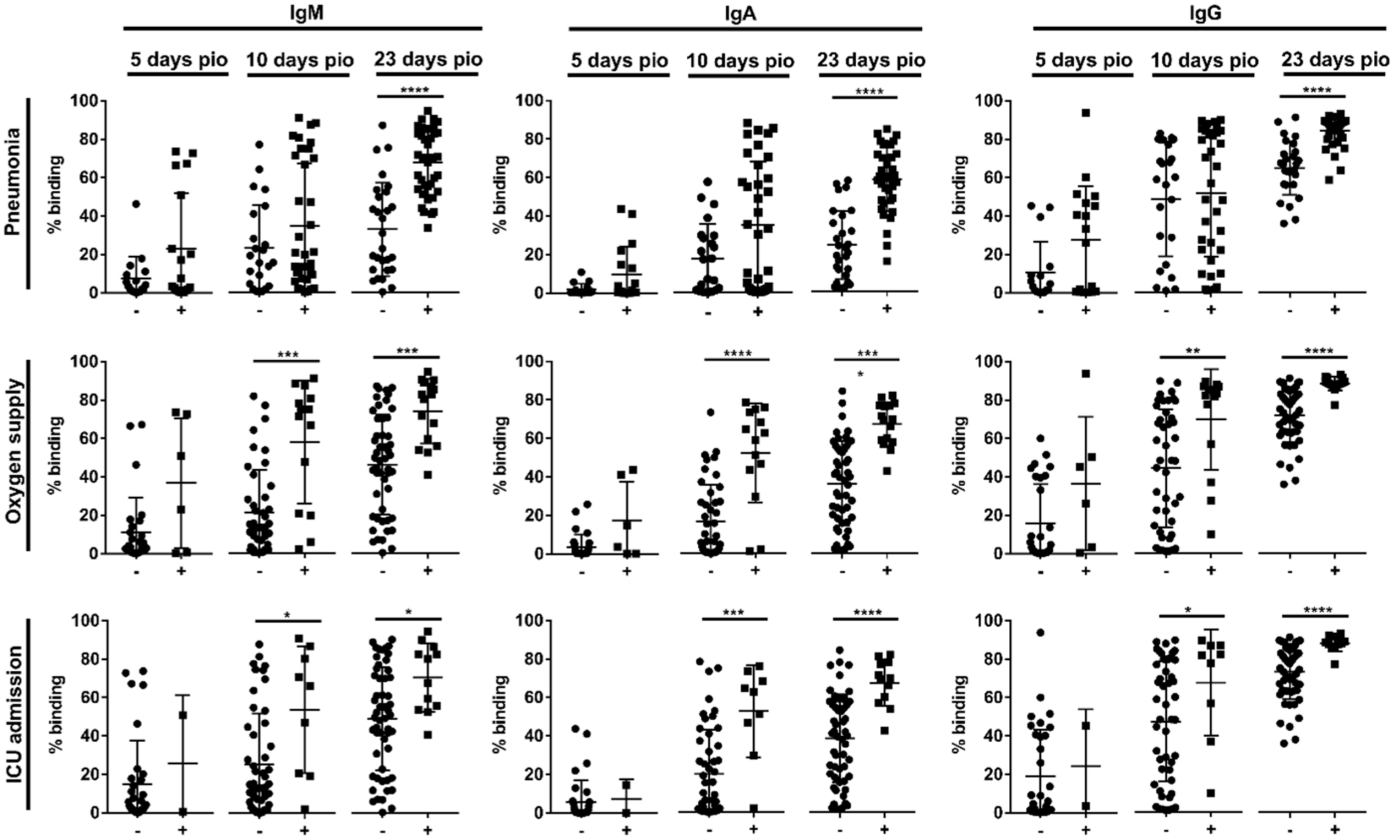
Association of specific IgM, IgA and IgG against full-length S protein with different clinical outcomes, pneumonia, oxygen supply requirement, and intensive care unit (ICU) admission. Negative and positive observations are denoted as - and +, respectively: pneumonia (negative:17 and positive:18 at median 5-days pio; negative:23 and positive:35 at median 10-days pio; negative:28 and positive:38 at median 23-days pio), oxygen supply requirement (negative:29 and positive:6 at median 5-days pio; negative:44 and positive:14 at median 10-days pio; negative:50 and positive:16 at median 23-days pio), and ICU admission (negative:33 and positive:2 at median 5-days pio; negative:49 and positive:9 at median 10-days pio; negative:54 and positive:12 at median 23-days pio). Symptomatic patients are grouped into mild (no pneumonia on chest radiographs), moderate (pneumonia on chest radiographs without hypoxia), and severe (pneumonia on chest radiographs with hypoxia) groups. ICU-admitted patients are a subset of severe patients (pneumonia on chest radiographs with hypoxia). For comparisons on pneumonia, patients without pneumonia include mild patients, while patients with pneumonia include moderate and severe patients. For comparisons on oxygen supply requirement, patients without oxygen supply requirement include mild and moderate patients, while patients with oxygen supply requirement include severe patients. For comparison on ICU admission, patients without ICU admission include mild, moderate and a subset of non-ICU-admitted severe patients, and patients with ICU admission include only the ICU-admitted severe patients. Hence, patients without oxygen supply requirement is a subset of the patients without ICU admission, and patients with ICU admission is a subset of the patients with oxygen supply requirement. Data are shown as mean ± SD of two independent experiments. Statistical analysis was carried out using Kruskal-Wallis tests, followed by *post hoc* Dunn’s multiple comparison tests. *p*-values for comparisons between the three severity groups are shown, where * indicates *p* ≤ 0.05, ** indicates *p* ≤ 0.01, *** indicates *p* ≤ 0.001, **** indicates *p* ≤ 0.0001.

Using a flow cytometry-based assay to detect antibodies against full-length S protein (SFB assay) (Goh et al., 2021a,b), we first examined if IgM, IgA and IgG against the ancestral wildtype (WT) S protein were associated with disease severity (*n* = 81; Supplementary Table S1) over the early course of infection, at time-points median 5-, 10-and 23-days post-illness onset (pio). At median 5-days pio, the antibody responses against S protein were low (Supplementary Figure 2). At median 10-and 23-days pio, IgM, IgA and IgG responses against S protein were associated with disease severity (Supplementary Figure 2), where patients with severe disease had higher IgM, IgA and IgG. We found that antibody responses against S protein were associated with pneumonia at median 23-days pio, while all three isotypes were associated with more severe clinical outcomes, requirement for supplemental oxygen and ICU admission, at a median of 10-days pio (Figure 1).

**Figure 2:**
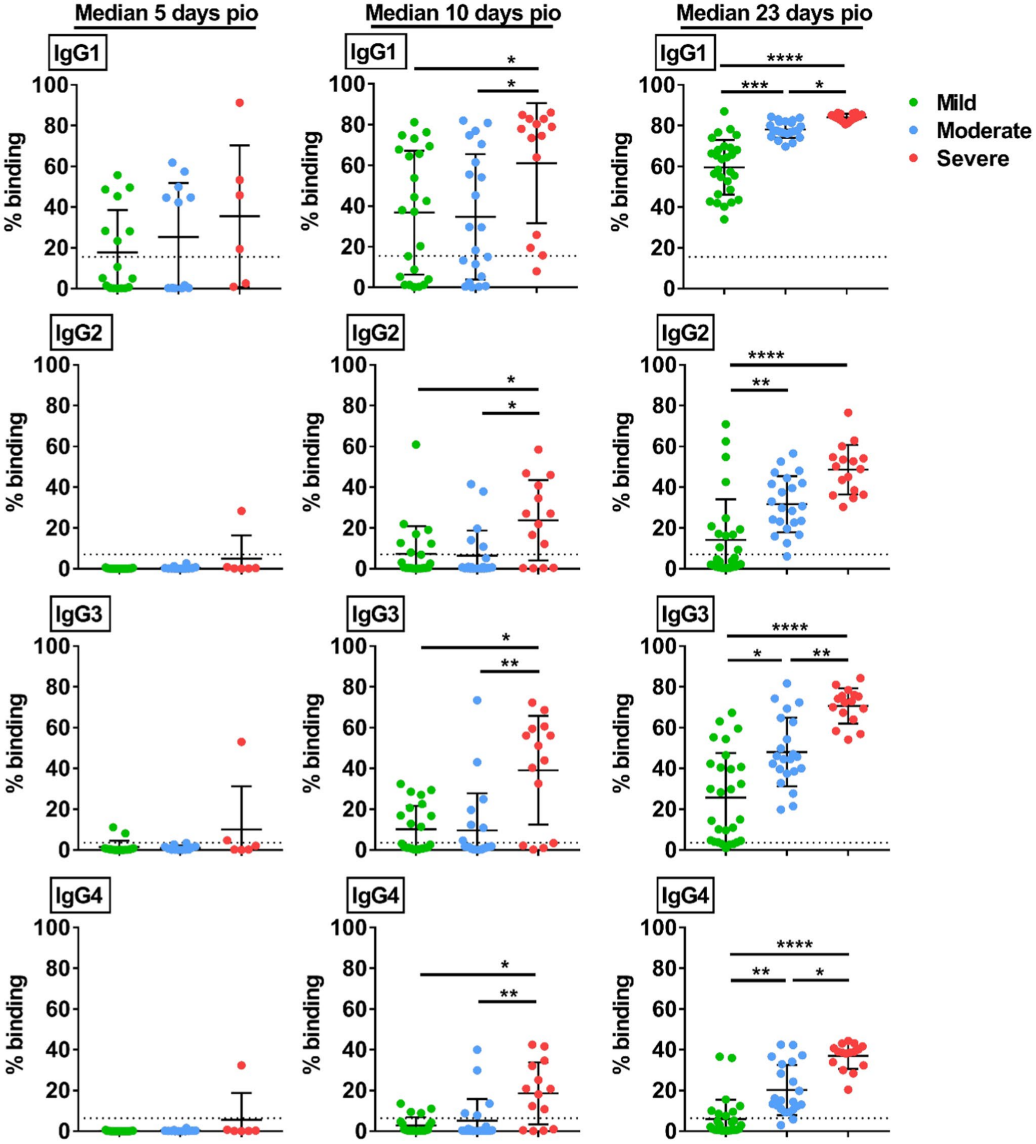
Specific IgG subclasses against full-length S protein. Plasma samples collected from 81 symptomatic unvaccinated COVID-19 patients at time-points median 5-days pio (mild, *n* = 17; moderate, *n* = 12; severe, *n* = 6), 10-days pio (mild, *n* = 23; moderate, *n* = 21; severe, *n* = 14) and 23-days pio (mild, *n* = 28; moderate, *n* = 22; severe, *n* = 16) were further screened for IgG subclasses, IgG1, IgG2, IgG3, and IgG4, against WT S protein. Data are shown as mean ± SD of two independent experiments. Statistical analysis was carried out using Kruskal-Wallis tests, followed by *post hoc* Dunn’s multiple comparison tests. Only *p*-values for comparisons between the three severity groups are shown, where * indicates *p* ≤ 0.05, ** indicates *p* ≤ 0.01, *** indicates *p* ≤ 0.001, **** indicates *p* ≤ 0.0001. Dotted lines indicate mean + 3SD of the healthy controls.

**Figure 3:**
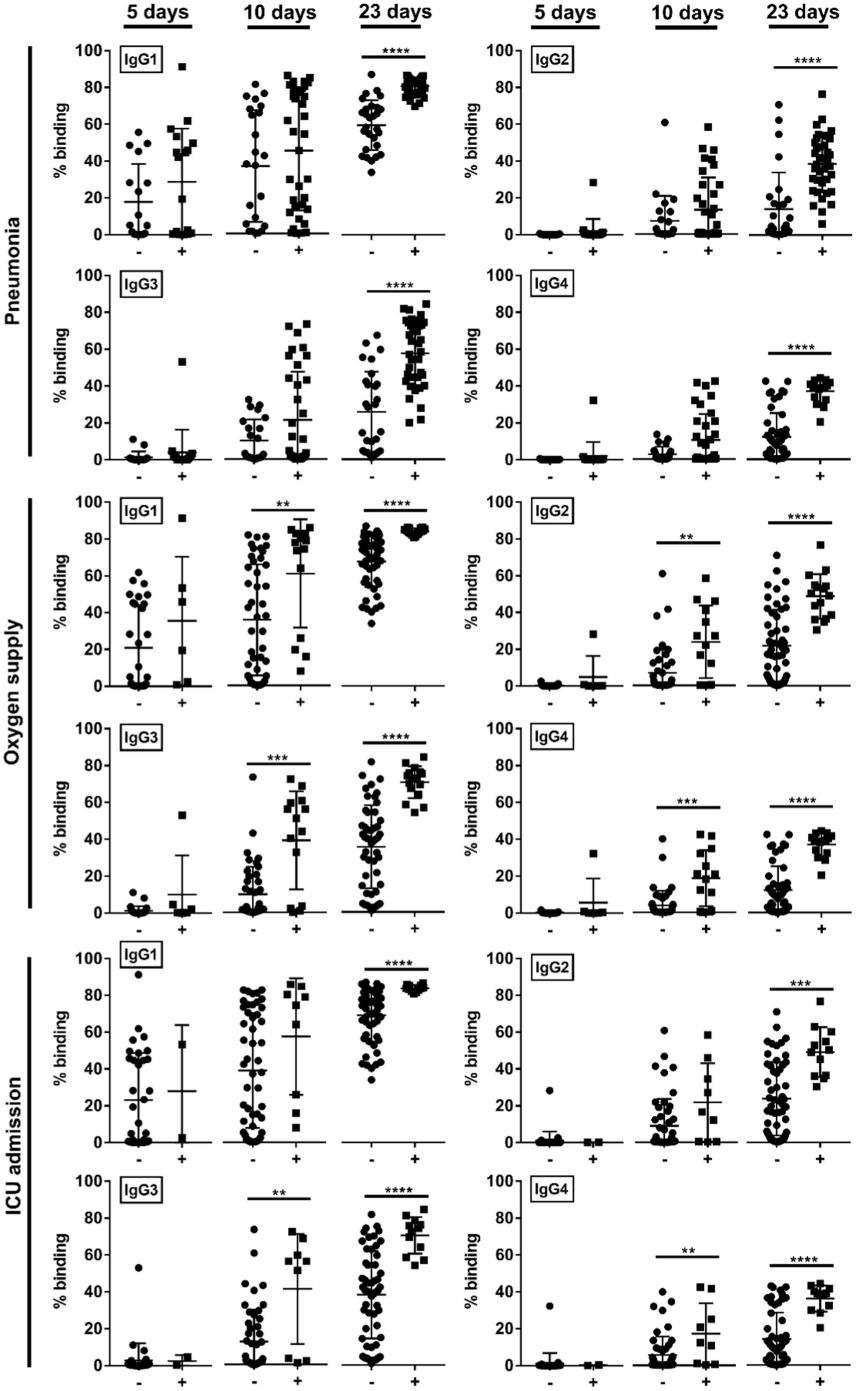
Association of specific IgG subclasses against full-length S protein with different clinical outcomes, pneumonia, oxygen supply requirement, and intensive care unit (ICU) admission. Negative and positive observations are denoted as - and +, respectively: pneumonia (negative:17 and positive:18 at median 5-days pio; negative:23 and positive:35 at median 10-days pio; negative:28 and positive:38 at median 23-days pio), oxygen supply requirement (negative:29 and positive:6 at median 5-days pio; negative:44 and positive:14 at median 10-days pio; negative:50 and positive:16 at median 23-days pio), and ICU admission (negative: 33 and positive: 2 at median 5-days pio; −: 49 and positive: 9 at median 10-days pio; negative:54 and positive:12 at median 23-days pio). Symptomatic patients are grouped into mild (no pneumonia on chest radiographs), moderate (pneumonia on chest radiographs without hypoxia), and severe (pneumonia on chest radiographs with hypoxia) groups. ICU-admitted patients are a subset of severe patients (pneumonia on chest radiographs with hypoxia). For comparisons on pneumonia, patients without pneumonia include mild patients, while patients with pneumonia include moderate and severe patients. For comparisons on oxygen supply requirement, patients without oxygen supply requirement include mild and moderate patients, while patients with oxygen supply requirement include severe patients. For comparison on ICU admission, patients without ICU admission include mild, moderate and a subset of non-ICU-admitted severe patients, and patients with ICU admission include only the ICU-admitted severe patients. Hence, patients without oxygen supply requirement is a subset of the patients without ICU admission, and patients with ICU admission is a subset of the patients with oxygen supply requirement. Data are shown as mean ± SD of two independent experiments. Statistical analysis was carried out using Kruskal-Wallis tests, followed by *post hoc* Dunn’s multiple comparison tests. *p*-values for comparisons between the three severity groups are shown, where ** indicates *p* ≤ 0.01, *** indicates *p* ≤ 0.001, **** indicates *p* ≤ 0.0001.

We then studied if the disease severity was associated with a particular IgG subclass. At median 5-days pio, IgG subclass responses were low (Figure 2). At median 10-and 23-days pio, where the IgG subclass responses were higher, we found an association between all IgG subclasses and the disease severity (Figure 2). We also observed an association between all subclasses and pneumonia, requirement for supplemental oxygen and ICU admission (Figure 3). Higher IgG1, IgG2, IgG3 and IgG4 were detected in patients with pneumonia at median 23-days pio, while the association between higher subclass responses and requirement for supplemental oxygen was observed as early as 10-days pio.

### IgG subclass skew is associated with disease severity

**Figure 4:**
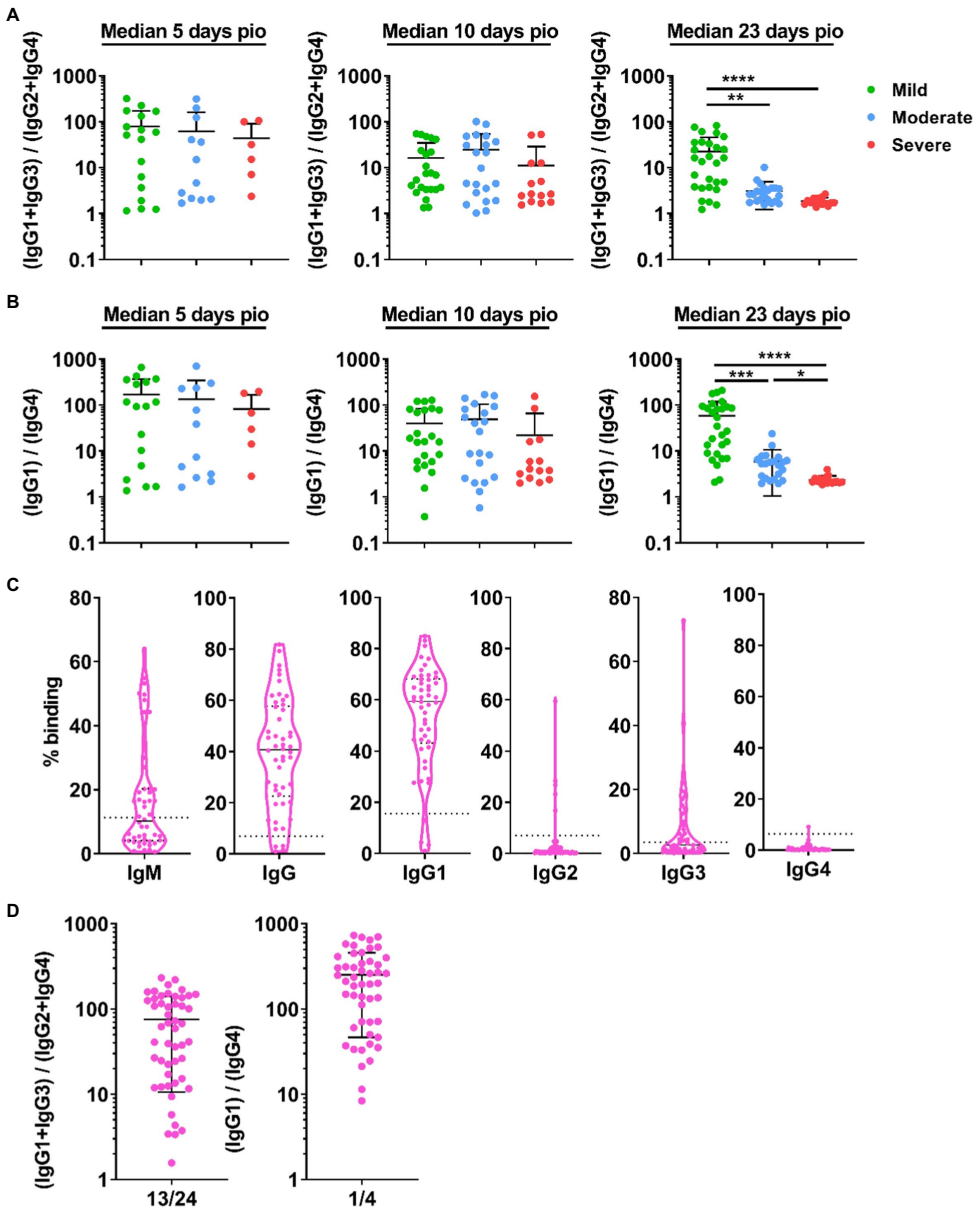
IgG subclass skew in symptomatic and asymptomatic COVID-19 patients. IgG1, IgG2, IgG3, and IgG4 response against WT S protein in plasma samples collected from 81 symptomatic unvaccinated COVID-19 patients at time-points median 5-days post-illness onset (pio) (mild, *n* = 17; moderate, *n* = 12; severe, *n* = 6), 10-days pio (mild, *n* = 23; moderate, *n* = 21; severe, *n* = 14) and 23-days pio (mild, *n* = 28; moderate, *n* = 22; severe, *n* = 16) were further analyzed. **(A)** Ratio of combined IgG1 and IgG3 response to combined IgG2 and IgG4response, and **(B)** ratio of IgG1 response to IgG4 response of the symptomatic patients are plotted. **(C)** Specific antibodies against full-length S protein in plasma samples collected from asymptomatic COVID-19 patients (*n* = 50) on the day of positive PCR confirmation. Dotted lines indicate mean + 3SD of the healthy controls. **(D)** Ratio of combined IgG1 and IgG3 response to combined IgG2 and IgG4 response and ratio of IgG1 response to IgG4 response of the asymptomatic unvaccinated patients are plotted. Data are shown as mean ± SD of two independent experiments. Statistical analysis was carried out using Kruskal-Wallis tests, followed by *post hoc* Dunn’s multiple comparison tests. *p*-values for comparisons between the three severity groups are shown, where * indicates *p* ≤ 0.05, ** indicates *p* ≤ 0.01, *** indicates *p* ≤ 0.001, **** indicates *p* ≤ 0.0001.

We investigated whether disease severity was associated with IgG subclass skew. To this end, we combined the IgG1 and IgG3 responses, and IgG2 and IgG4 responses, and computed the ratio of the combined IgG1 and IgG3 response to the combined IgG2 and IgG4 responses (Figure 4A), abbreviated as 13/24 ratio. A ratio higher than 1 indicates IgG subclass response that is skewed towards a more dominant IgG1 and IgG3 response. Comparing the 13/24 ratio across the different severity groups, we found that the 13/24 ratio was associated with disease severity at median 23-days pio, where the skew towards IgG1 and IgG3 response was greater in patients with mild disease than patients with moderate and severe disease. The mild group has a mean 13/24 ratio of 22.39, compared with 1.56 and 1.38 in the moderate and severe groups, respectively. We also computed the ratio of the IgG1 response to the IgG4 response, abbreviated as 1/4 ratio (Figure 4B). Interestingly, the association between the 1/4 ratio and disease severity was more pronounced – the 1/4 ratio was significantly higher in the mild group (58.88) than the moderate group (5.88), which, in turn, was significantly higher than the severe group (2.36).

We then investigated if patients with no symptom also have an IgG subclass skew. Asymptomatic patients (*n* = 50; Supplementary Table S1) developed specific antibodies, and IgG1 is the dominant IgG subclass response (Figure 4C). Indeed, the asymptomatic patients also had an IgG subclass response skewed towards a dominant IgG1 and IgG3 response (mean 13/24 ratio: 75.41; mean 1/4 ratio: 252.1, Figure 4D).

### IgG subclass over the course of infection

**Figure 5:**
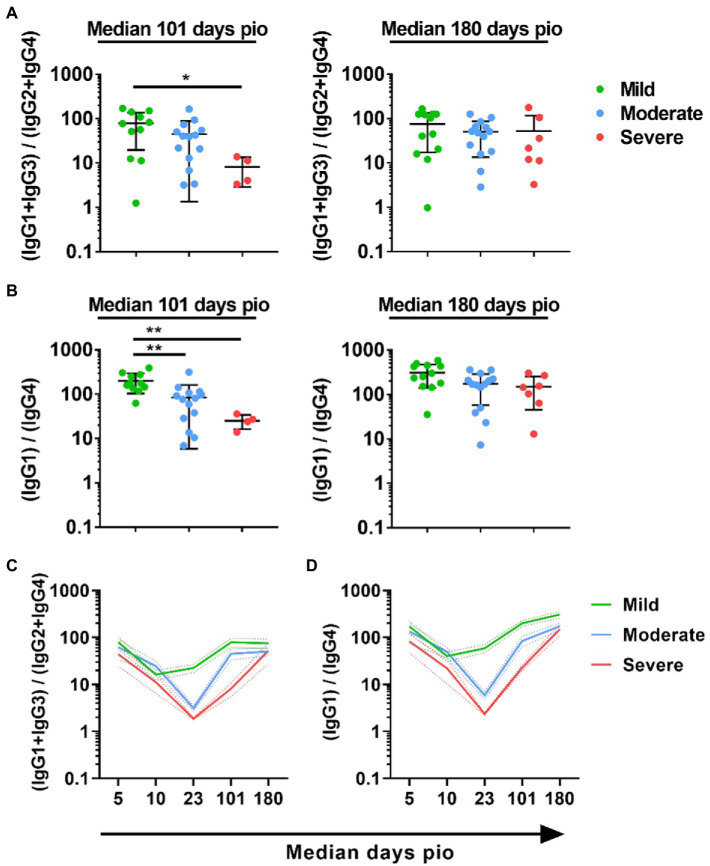
Longitudinal examination of IgG subclass skew in symptomatic and asymptomatic COVID-19 patients. IgG1, IgG2, IgG3, and IgG4 response against WT S protein in plasma samples collected from 81 symptomatic unvaccinated COVID-19 patients at time-points median 101 (mild, *n* = 11; moderate, *n* = 14; severe, *n* = 4) and 180 (mild, *n* = 17; moderate, *n* = 12; severe, *n* = 6) were further analyzed. **(A)** ratio of combined IgG1 and IgG3 response to combined IgG2 and IgG4 response, 13/24, **(B)** Ratio of IgG1 response to IgG4 response, 1/4, of the symptomatic patients are plotted. Longitudinal profiles of **(C)** ratio of combined IgG1 and IgG3 response to combined IgG2 and IgG4 response, 13/24, and **(D)** ratio of IgG1 response to IgG4 response, 1/4, up to median 180-days pio in COVID-19 patients, stratified by disease severity outcome. Data are shown as mean ± SD of two independent experiments. Statistical analysis was carried out using Kruskal-Wallis tests, followed by *post hoc* Dunn’s multiple comparison tests. *p*-values for comparisons between the three severity groups are shown, where * indicates *p* ≤ 0.05, ** indicates *p* ≤ 0.01.

To understand if IgG subclass skew was sustained over the course of infection, we examined the subclass response at median 101-and 180-days pio. At these time-points, all patients, including the severe patients, have recovered from the disease. All patients retained substantial IgG1 response, and IgG1 remained the dominant subclass (Supplementary Figure 3). IgG2, IgG3 and IgG4 responses were low for all severity groups at both time-points. When we compared the 13/24 and 1/4 ratios across the severity groups, the trend, where the ratio was higher with the mild severity group than the moderate and severe severity group, was still sustained at median 101-days pio (Figures 5A,B). However, there was no significant difference at median 180-days pio. Comparing between the different time-points, we observed that both 13/24 and 1/4 ratios were the lowest at median 10-days pio for patients with mild symptoms (Figures 5C,D). Patients with moderate and severe symptoms exhibited the lowest 13/24 and 1/4 ratios at median 23-days pio. Both 13/24 and 1/4 ratios increased over time, suggesting that IgG subclass skew towards a greater IgG1 and IgG3 dominance increased over time, in line with clinical recovery.

### IgG subclass skew in vaccine breakthrough Delta infections over time

Having found that acute severity and eventual clinical recovery is associated with IgG subclass skew, we proceeded to examine if this also stands true for vaccine breakthrough infections. Vaccine breakthrough infections are defined as infections that occur at least 2 weeks following a two-dose primary vaccination. The cases (*n* = 118, Supplementary Table S1) are identified as Delta vaccine breakthrough infections *via* epidemiological data (Wang et al., 2021) and direct sequencing. Out of the 118 cases, there were 104 cases with mild symptoms and 14 cases with moderate symptoms. None had severe symptoms.

**Figure 6:**
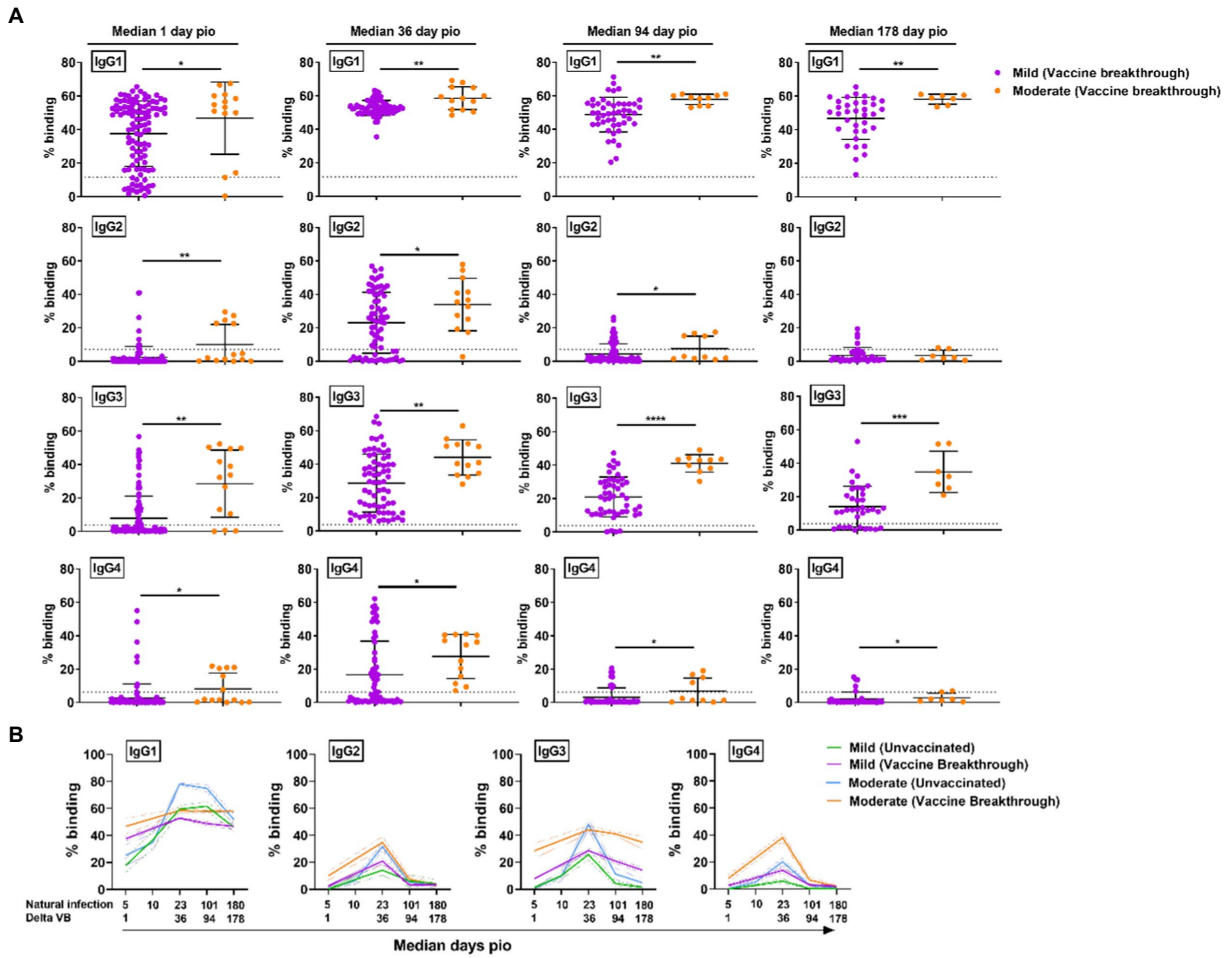
Specific antibodies against full-length Delta S protein in vaccine breakthrough infections at median 1, 36, 94 and 178-days pio. **(A)** Plasma samples collected from symptomatic vaccinated patients at time-points median 1 (mild, *n* = 104; moderate, *n* = 14), 36 (mild, *n* = 70; moderate, *n* = 13), 94 (mild, *n* = 48; moderate, *n* = 10), and 178 (mild, *n* = 36; moderate, *n* = 7) were analysed for antibody response against Delta S protein. **(B)** Line plot representation to show kinetics of antibody responses over time. Data are shown as mean ± SD of two independent experiments, with dotted lines indicating mean + 3SD of healthy donors. Statistical analysis was carried out using Kruskal-Wallis tests, followed by *post hoc* Dunn’s multiple comparison tests. Only *p*-values for comparisons between the three severity groups are shown, where * indicates *p* ≤ 0.05, ** indicates *p* ≤ 0.01. For line plots, data were represented as mean + SEM. *** indicates *p* ≤ 0.001 and **** indicates *p* ≤ 0.0001.

**Figure 7:**
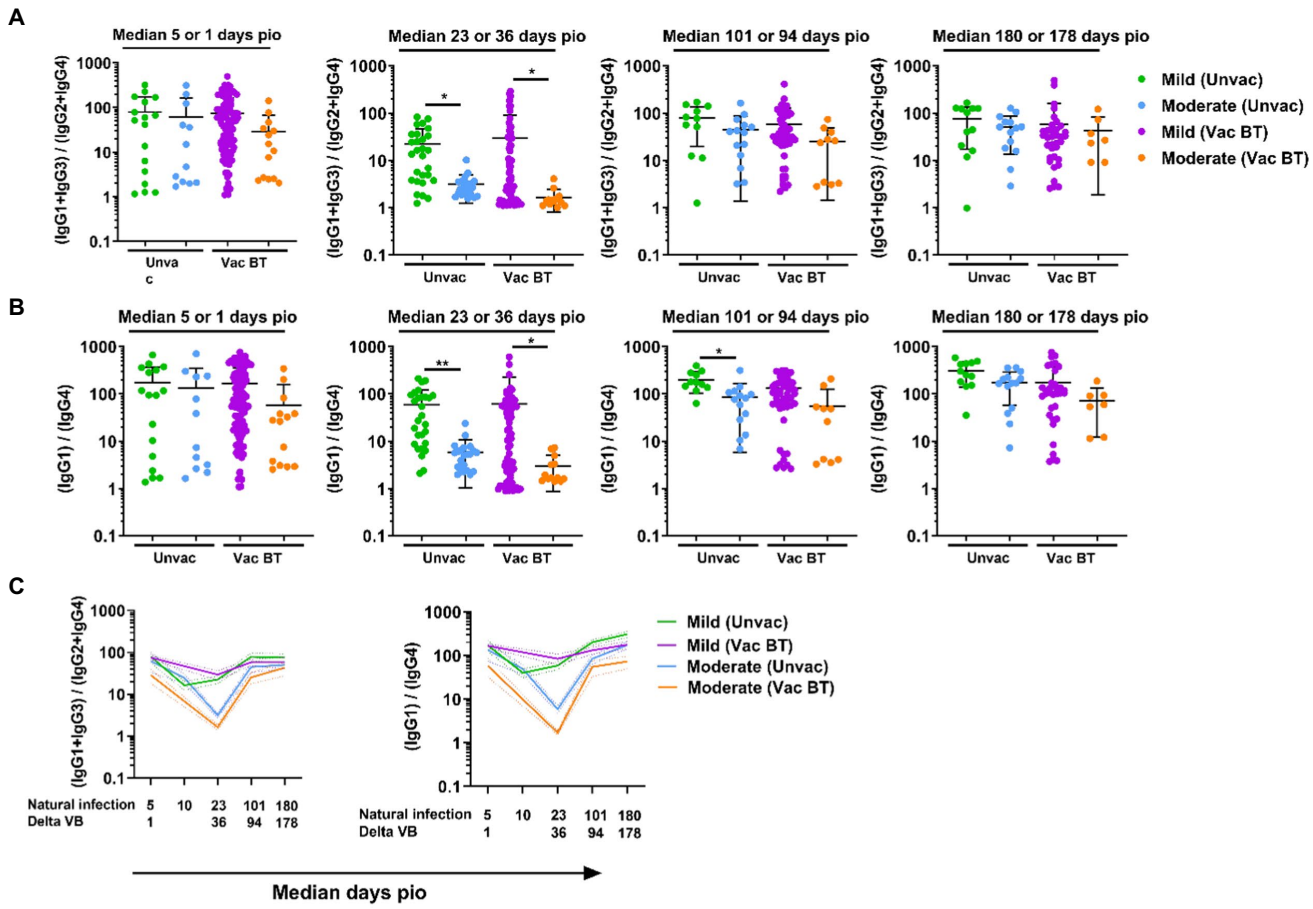
Longitudinal examination of IgG subclass skew in vaccine breakthrough infections. IgG1, IgG2, IgG3, and IgG4 response against Delta S protein in plasma samples collected from 118 symptomatic vaccinated patients at time-points median 1 (mild, *n* = 104; moderate, *n* = 14), 36 (mild, *n* = 70; moderate, *n* = 13), 94 (mild, *n* = 48; moderate, *n* = 10), and 178 (mild, *n* = 36; moderate, *n* = 7) were analyzed. **(A)** Ratio of combined IgG1 and IgG3 response to combined IgG2 and IgG4 response, 13/24, and **(B)** ratio of IgG1 response to IgG4 response, 1/4, of the symptomatic patients are plotted. Longitudinal profiles of **(C)** ratio of combined IgG1 and IgG3 response to combined IgG2 and IgG4 response, 13/24, and ratio of IgG1 response to IgG4 response, 1/4, up to median 180-days pio in COVID-19 patients, stratified by disease severity outcome. Data are shown as mean ± SD of two independent experiments. Statistical analysis was carried out using Kruskal-Wallis tests, followed by *post hoc* Dunn’s multiple comparison tests. *p*-values for comparisons between the three severity groups are shown, where * indicates *p* ≤ 0.05, ** indicates *p* ≤ 0.01. Data from unvaccinated individuals (Figures 4, 5) were replotted for comparison purposes. For line plots, data were represented as mean + SEM. Unvac: unvaccinated; Vac BT: Vaccine breakthrough.

As the vaccine breakthrough infections in this study are Delta vaccine breakthrough infections, we analyzed the antibody response against the Delta S protein using the SFB assay with cells expressing the full-length Delta S protein. We found that, similar to infections in unvaccinated individuals, higher IgG subclasses were also associated with disease progression in the Delta vaccine breakthrough infections (Figure 6A). Vaccinated individuals with moderate symptoms had significantly higher levels of all four IgG subclasses than vaccinated individuals with mild symptoms as early as median 1-day pio (Figures 6A,B). We did not observe any association of disease progression with either 13/24 (Figure 7A) or 1/4 (Figure 7B) ratios at median 1-day pio. However, at median 36-days pio, the 13/24 ratio was associated with disease progression, where the skew towards IgG1 and IgG3 response was greater in vaccinated patients with mild disease than vaccinated patients with moderate disease. Similar to the unvaccinated infected individuals, the 13/24 and 1/4 ratios increased over time with disease resolution (Figure 7C). By median 94-days pio, the vaccine breakthrough cases have resolved their infections, and there was no significant difference in the 13/24 and 1/4 ratios between the severity groups. It is worth noting that, while IgG2 and IgG4 profiles remained fairly similar for both mild and moderate infections in both vaccinated and unvaccinated individuals, IgG1 and IgG3 profiles differed between the vaccinated and unvaccinated individuals (Figure 6B). In particular, unvaccinated individuals with moderate symptoms had higher peak IgG1 levels than vaccinated individuals with moderate symptoms. In addition, waning of IgG1 and IgG3 levels (between 36-178-days pio) was more subtle following infection in vaccinated individuals, compared with unvaccinated individuals. Despite the differences in IgG subclasses, the 13/24 and 1/4 ratio profiles were similar (Figure 7C), highlighting that greater 13/24 and 1/4 ratios might also be associated with clinical recovery in vaccine breakthrough Delta infections.

## Discussion

The understanding of the immune mechanisms leading to severe disease, and the identification of biomarkers of disease severity and/or resolution of the infection remains limited and inadequate. In this study, we found that S protein antibody levels were strongly associated with disease severity, in line with other studies (Amrun et al., 2020; Long et al., 2020; Okba et al., 2020). A few studies have reported an association between IgG1 and IgG3 with severe disease and suggested its potential contribution in disease progression (Yates et al., 2020; Chakraborty et al., 2021; Luo et al., 2021). High levels of afucosylated antibodies, mainly IgG1, were found to associate with progression from mild to severe disease in non-vaccinated individuals (Chakraborty et al., 2020; Larsen et al., 2021). Afucosylated IgG1 complexed to the S-protein binds to FcRIIa and/or FcγRIIIa on monocyte/macrophage to induce the release of pro-inflammatory cytokines (Chakraborty et al., 2020; Hoepel et al., 2021; Larsen et al., 2021). However, in this study, we have observed an association between antibody levels and disease severity for all IgG subclasses, and not solely IgG1 and IgG3. More importantly, the comparison of the ratio of IgG1 and IgG3 to IgG2 and IgG4 (abbreviated as 13/24) or the ratio of IgG1 to IgG4 (abbreviated as 1/4) revealed that SARS-CoV-2 infections were associated with IgG subclass skew. Both 13/24 and 1/4 ratios were associated with disease severity, where a smaller skew towards IgG1 and IgG3 were associated with severe disease. The IgG subclass skew was also found in asymptomatic patients. Both 13/24 and 1/4 ratios were high in asymptomatic patients, at levels comparable or higher than patients with mild symptoms. It has been shown that asymptomatic patients have a highly functional virus-specific immune response, comparable to symptomatic patients (Le Bert et al., 2021). This further supported that a dominant IgG1 and IgG3 antibody response might be important in controlling symptom progression.

One of the main findings in this study is that the IgG subclass skew was also observed in vaccine breakthrough infections during the Delta variant wave. Despite the huge success of the WT S-protein-based mRNA vaccines, vaccine breakthrough infections occur. Waning of antibody response after full primary vaccination and the emergence of immune escape variants such as Delta and Omicron are some of the contributory factors. We found that, similar to unvaccinated individuals, there was also an IgG subclass skew against Delta S protein in these Delta vaccine breakthrough infections. In unvaccinated individuals, significant differences in antibody levels were only observed at later time-points, with more pronounced differences at median 23-days pio. It is worth noting that, at day 101 and 180 pio, the levels of IgG2, IgG3 and IgG4 were low, while the levels of IgG1 remains comparatively high. This is in line with observations in dengue viral infection (Nascimento et al., 2018), where only IgG1 but not IgG3 are detectable at 6 months pio. In contrast, subtle waning in both IgG1 and IgG3 levels was observed in vaccine breakthrough infections. Vaccine-induced antibody levels are reported to be more stable and decreasing at a slower rate in previously infected individuals, compared with individuals with no prior infections (Wei et al., 2021). Whether the subtle waning of infection-induced IgG1 and IgG3 levels in vaccine breakthrough infections is similar to the waning of vaccine-induced antibody levels in previously infected individuals and whether both offer similar protection remains to be determined. Despite the differences in IgG subclasses response, the 13/24 and 1/4 ratio profiles were similar in Delta vaccine breakthrough infections, as compared with natural WT infections. A lower IgG subclass skew toward IgG1 and IgG3 was also associated with disease progression at the earlier stages of these Delta vaccine breakthrough infections, where patients with moderate disease have a lower IgG subclass skew toward IgG1 and IgG3, compared to patients with mild disease. This further affirmed that the lower skew towards IgG1 and IgG3 is associated with disease progression, regardless of vaccination status, and may also be applicable to different variants.

While we observed an association between lower IgG subclass skew towards 13/24 and 1/4 ratios and disease severity at earlier time-points (median 23-days and 36-days pio in unvaccinated and vaccinated individuals respectively), the differences in 13/24 and 1/4 ratios between the severity groups became less pronounced over time. There were no significant differences in the IgG subclasses ratios between the severity groups by median 180-days and 178-days pio in unvaccinated and vaccinated individuals, respectively. In our cohort where all patients eventually recovered from the disease, our findings shows that an immune environment skewed towards IgG1 and IgG3 is favorable and may be crucial to clinical recovery. This is in agreement with other studies, where IgG3 has been identified as key isotype for neutralization capacity in convalescent plasma (Kober et al., 2022) and engineered monoclonal IgG3 antibodies have demonstrated superior neutralization capacity than the other IgG subclasses (Kallolimath et al., 2021). Taken together, vaccines that can induce an antibody response with a stronger skew toward IgG1 and IgG3 may offer better protection, potentially due to superior neutralization capacity. In contrast, in the acute phase of infection, an immune environment with robust IgG2 and IgG4 response may contribute the disease progression. IgG2 and IgG4 have been hypothesized to mediate either viral infection enhancement or disease enhancement (Liu et al., 2019; Arvin et al., 2020; de Alwis et al., 2020). IgG2 and IgG4 may functionally block the production of mild disease-associated interferon-stimulated gene-expressing cells and dampen cellular responses to interferons (Combes et al., 2021). The composition of the induced antibody response might be as important as the absolute amount of the antibody response in disease control.

This study has two main limitations. First, we were not able to acquire samples from non-vaccinated individuals infected with the Delta strain or vaccine breakthrough infection with the ancestral Wuhan wildtype strain. This was due to the epidemiological situation in Singapore then – at the start of this part of the study (April 2021), over 80% of the adult population in Singapore was vaccinated and the Delta variant was the dominant circulating strain (Wang et al., 2021). Second, we have not investigated the level and the proportion of afucosylated antibodies against the S protein in the total antibody response in our cohort. It is possible that there may also be a higher proportion of afucosylated antibodies in the early stages of initial WT and vaccine breakthrough Delta infections and the high proportion of afucosylated antibodies may contribute to disease progression.

In conclusion, our findings demonstrate that SARS-CoV-2 infections led to IgG subclass skew towards IgG1 and IgG3 over IgG2 and IgG4. We show that a greater presence of Th2-associated IgG2 and IgG4 (resulting in a smaller skew in IgG subclass imbalance towards Th1-associated IgG1 and IgG3) in the early phase of infection is associated with severe disease, while a stronger skew towards Th1-associated IgG1 and IgG3 may be important in controlling disease progression and clinical recovery. This is supported by data showing that an initial blunted interferon response and heightened T-helper 2 inflammatory response is associated with severe disease (Baker et al., 2022), and is line with other studies (Roncati et al., 2020; Gibellini et al., 2022), showing that Th2 polarization, rather than Th1, is predictive of severe disease. A recent study showed that an imbalance between Th1 and Th2 cytokine release was associated with high susceptibility to COVID-19 (Fahrner et al., 2022). Th2/Th1 imbalance was higher in patients at admission that do not survive COVID-19 (Pavel et al., 2021) and a high Th2 response was associated with a fatal disease outcome (Gil-Etayo et al., 2021). Th2 responses, rather than Th1 responses, were associated with intensive care (Roncati et al., 2020). A robust Th1 response may restrain infection while a Th2 response may limit elimination of infected cells and delay recovery (Fahrner et al., 2022). IgG subclass analysis may be used as a biomarker of Th1/Th2 imbalance for prediction of disease progression. Importantly, the findings are clinically relevant, especially in the current COVID-19 landscape. A smaller IgG subclass skew IgG1 and IgG3 may also contribute to disease progression in vaccine breakthrough infections by emerging variants such as Delta.

## Data availability statement

The raw data supporting the conclusions of this article will be made available by the authors, without undue reservation.

## Ethics statement

The studies involving human participants were reviewed and approved by National Healthcare Group Domain Specific Review Board. The patients/participants provided their written informed consent to participate in this study.

## Author contributions

YG conceptualized study, designed, and conducted the experiments, analyzed the data, and wrote the manuscript. S-WF, SA, PH, and CY-PL designed and conducted the experiments, analyzed the data, and wrote the manuscript. BY, MI-CC, PT, SK, SP, S-YT, LS, Y-SL, and DL designed and supervised sample collection. LN and LR conceptualized study and wrote the manuscript. All authors revised and approved the final version of the manuscript.

## Funding

This research was supported by Biomedical Research Council (BMRC), the A*ccelerate GAP-funded project (ACCL/19-GAP064-R20H-H) from Agency of Science, Technology and Research (A*STAR), National Medical Research Council (NMRC) COVID-19 Research fund (COVID19RF-001, COVID-19RF-007, and COVID-19RF-60), and A*STAR COVID-19 Research funding (H/20/04/g1/006).

## Conflict of interest

A patent application for the SFB assay has been filed (Singapore patent #10202009679P: A Method of Detecting Antibodies and Related Products).The authors declare that the research was conducted in the absence of any commercial or financial relationships that could be construed as a potential conflict of interest.

## Publisher’s note

All claims expressed in this article are solely those of the authors and do not necessarily represent those of their affiliated organizations, or those of the publisher, the editors and the reviewers. Any product that may be evaluated in this article, or claim that may be made by its manufacturer, is not guaranteed or endorsed by the publisher.
