# Supplementary material for: Conserved longitudinal alterations of anti-S-protein IgG subclasses in disease progression in initial ancestral Wuhan and vaccine breakthrough Delta infections
Source: Front Microbiol. 2022 Nov 22;13:1043049. doi: 10.3389/fmicb.2022.1043049 (PMC9723332; doi:10.3389/fmicb.2022.1043049)
Supplement: Supplementary file 1 [file Table_1.DOCX]

Table S1. Demographic and clinical information of symptomatic COVID-19 patients, asymptomatic COVID-19 patients, healthy donors, recovered SARS and seasonal human CoV patients.

| **Symptomatic unvaccinated COVID-19 patients** | **Patients (N=81)** |
| --- | --- |
| **Age, Mean years (SD)** | 45 (13) |
| Gender, n (%) |  |
| Male | 48 (59.3%) |
| Female | 33 (40.7%) |
| Ethnicity, n (%) |  |
| Chinese | 68 (84.0%) |
| Others | 13 (16.0%) |
| Co-morbidities, n (%) |  |
| Diabetes | 7 (8.6%) |
| Hypertension | 15 (18.5%) |
| Others | 11 (13.6%) |
| Vital signs at admission |  |
| Temperature, Mean °C, (SD) | 37.7 (0.9) |
| Heart rate, Mean beats/minute (SD) | 91.4 (16.6) |
| Respiratory rate, Mean rate per minute (SD) | 18.4 (1.9) |
| Diastolic blood pressure, Mean mmHg (SD) | 97.5 (2.4) |
| Systolic blood pressure, Mean mmHg (SD) | 132.2 (18.5) |
| Oxygen saturation, Mean % (SD) | 77.8 (15.2) |
| Laboratory findings |  |
| Haemoglobin, Mean g/dL (SD) | 13.8 (1.6) |
| Haematocrit, Mean % (SD) | 40.8 (4.6) |
| Platelets, Mean x 10^9^/L (SD) | 194.8 (69.8) |
| White blood cells, Mean x 10^9^/L (SD) | 5.3 (3.0) |
| Lymphocytes, Mean x 10^9^/L (SD) | 1.2 (0.6) |
| Neutrophils, Mean x 10^9^/L (SD) | 4.3 (7.7) |
| Monocytes, Mean x 10^9^/L (SD) | 0.6 (1.1) |
| C-reactive protein, Mean mg/L (SD) | 37.4 (55.7) |
| Creatinine, Mean μmol/L (SD) | 75.0 (45.3) |
| Lactate dehydrogenase, Mean U/L (SD) | 514.3 (298.4) |
| Alanine aminotransferase, Mean U/L (SD) | 34.6 (28.1) |
|  |  |
| **Asymptomatic unvaccinated COVID-19 patients** | **Patients (N=50)** |
| Age, Mean years (SD) | 40 (12) |
| Gender, n (%) |  |
| Male | 41 (82.0%) |
| Female | 9 (18.0%) |
| Ethnicity, n (%) |  |
| Chinese | 5 (10.0%) |
| Others | 45 (90.0%) |
| Co-morbidities, n (%) |  |
| Diabetes | 6 (12.0%) |
| Hypertension | 10 (20.0%) |
| Others | 12 (24.0%) |
|  |  |
| **Symptomatic Delta Vaccine breakthrough infections** | **Patients (N=118)** |
| Interval between last vaccine dose and infection, median days | 92 |
| Age, Mean years (SD) | 45 (13) |
| Gender, n (%) |  |
| Male | 48 (59.3%) |
| Female | 33 (40.7%) |
| Ethnicity, n (%) |  |
| Chinese | 68 (84.0%) |
| Others | 13 (16.0%) |
| Co-morbidities, n (%) |  |
| Diabetes | 7 (8.6%) |
| Hypertension | 15 (18.5%) |
| Others | 11 (13.6%) |
| Clinical severity at initial infection |  |
| No pneumonia (mild), n (%) | 104 (88.1%) |
| Pneumonia, without hypoxia (moderate), n (%) | 14 (11.9%) |
| Pneumonia, with hypoxia (severe) n (%) | 0 (0) |
|  |  |
| **Healthy donors** | **N=22*** |
| Age, Mean years (SD) | 45 (13) |
| Gender, n (%) |  |
| Male | 9 (40.9%) |
| Female | 12 (54.5%) |
|  |  |

* Information of one donor unknown. **Ministry of Health. SARS: Severe Acute Respiratory Syndrome Coronavirus; human CoV: human coronavirus
