# Supplementary figures and images for: Conserved longitudinal alterations of anti-S-protein IgG subclasses in disease progression in initial ancestral Wuhan and vaccine breakthrough Delta infections
Source: Front Microbiol. 2022 Nov 22;13:1043049. doi: 10.3389/fmicb.2022.1043049 (PMC9723332; doi:10.3389/fmicb.2022.1043049)

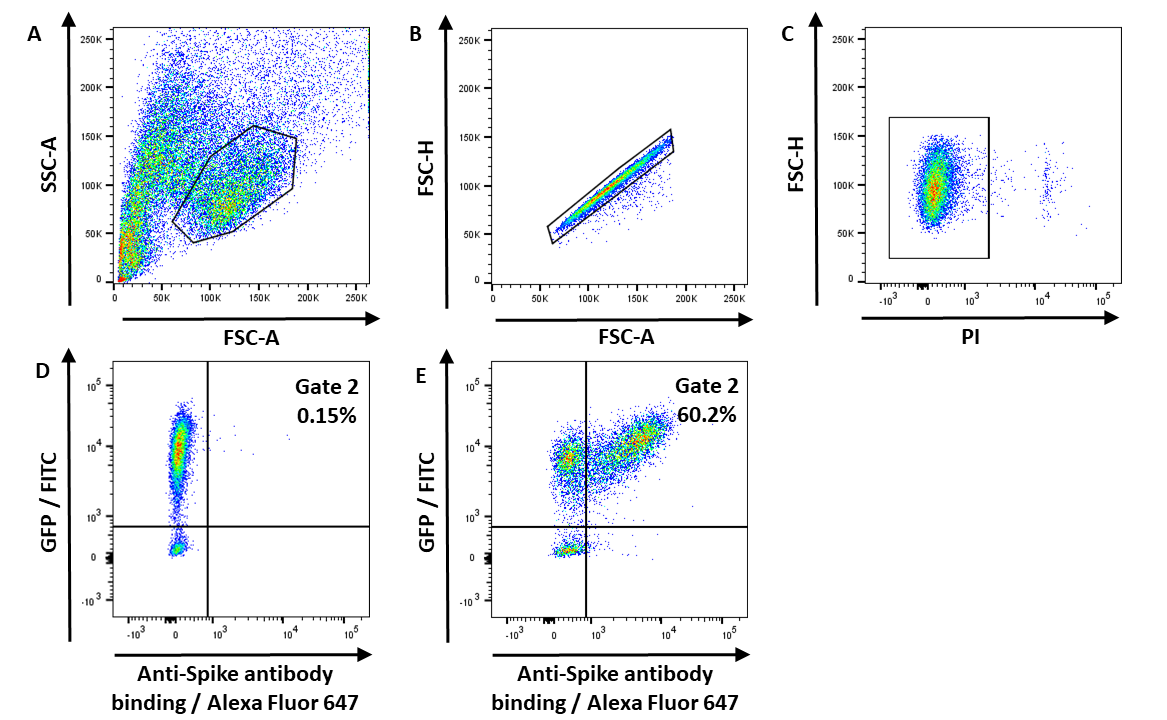

Supplement: Supplementary file 2 [file Image_1.TIF]

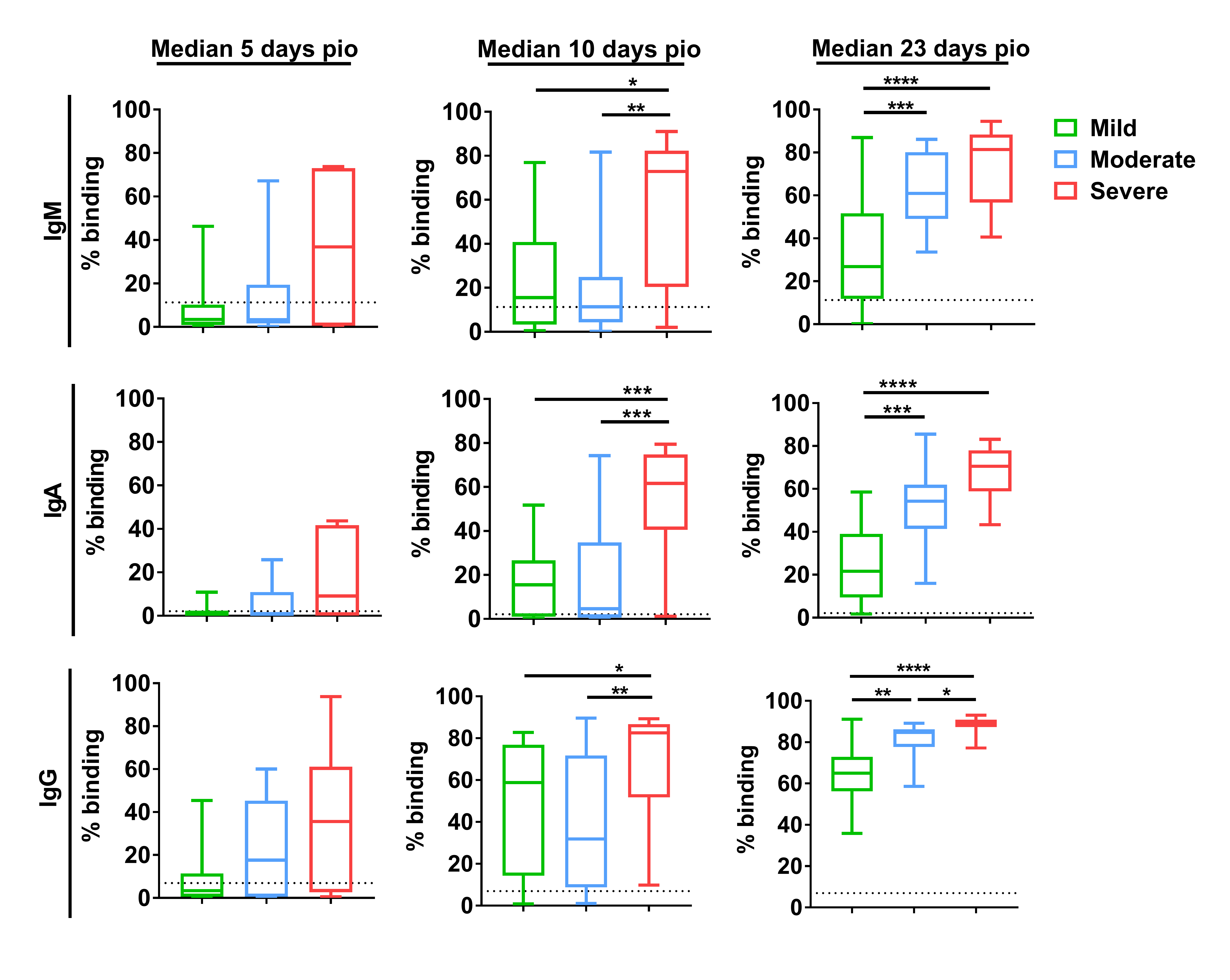

Supplement: Supplementary file 3 [file Image_2.TIF]

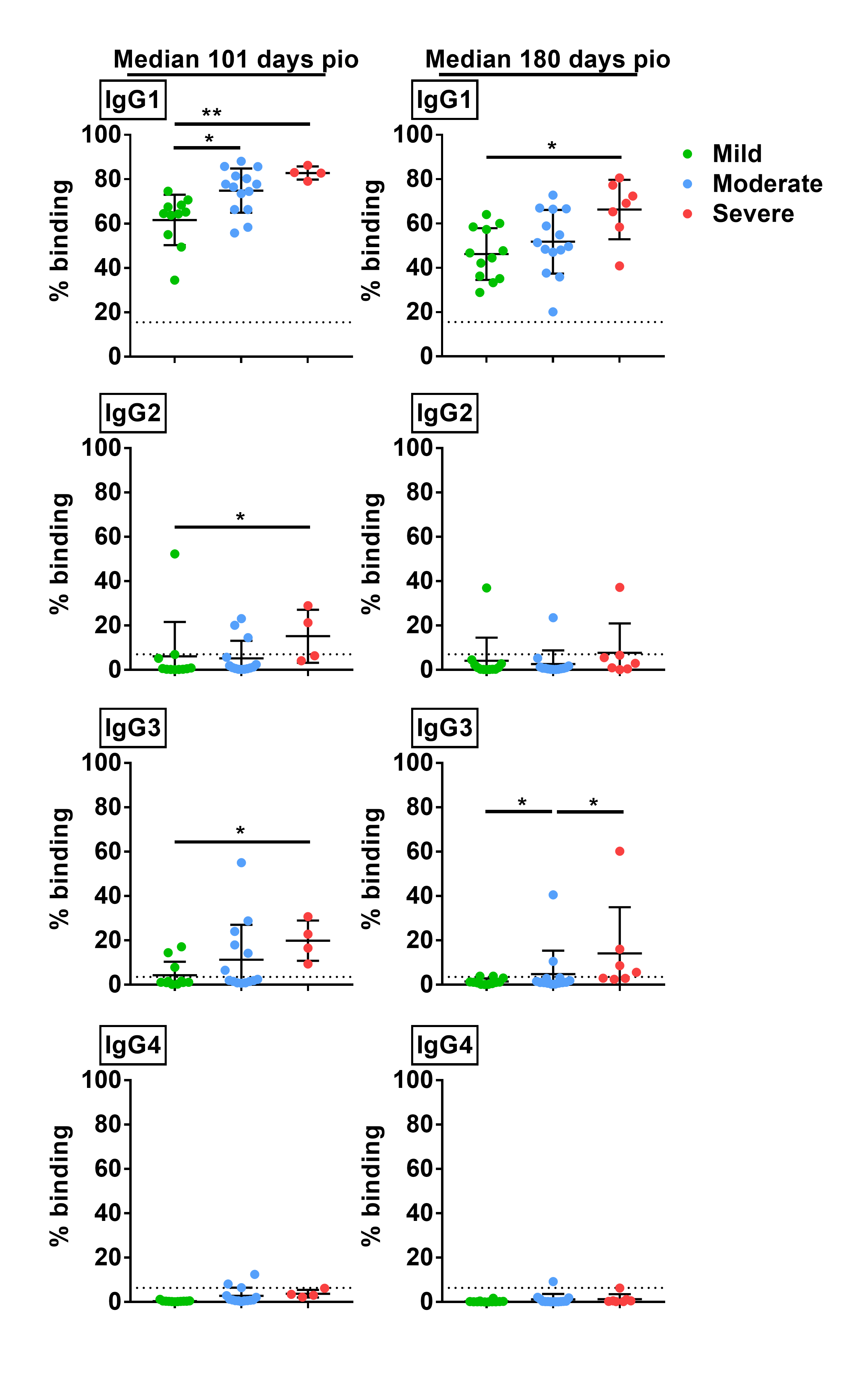

Supplement: Supplementary file 4 [file Image_3.TIF]
